# Biomechanical evaluation of shape-memory alloy staples for internal fixation—an in vitro study

**DOI:** 10.1186/s40634-016-0055-3

**Published:** 2016-08-30

**Authors:** QiCai Jason Hoon, Matthew H. Pelletier, Chris Christou, Kenneth A. Johnson, William R. Walsh

**Affiliations:** 1 Surgical and Orthopaedic Research Laboratories (SORL), Prince of Wales Clinical School, University of New South Wales, Sydney, 2031 NSW Australia; 2 Faculty of Veterinary Science, University of Sydney, Sydney, 2006 NSW Australia

**Keywords:** Nitinol, Staple, Shape memory, Internal fixation, Biomechanical

## Abstract

**Background:**

The field of orthopaedics is a constantly evolving discipline. Despite the historical success of plates, pins and screws in fracture reduction and stabilisation, there is a continuing search for more efficient and improved methods of fracture fixation. The aim of this study was to evaluate shape-memory staples and to compare them to a currently used implant for internal fracture fixation. Multi-plane bending stability and interfragmentary compression were assessed across a simulated osteotomy using single and double-staple fixation and compared to a bridging plate.

**Methods:**

Transverse osteotomies were made in polyurethane blocks (20 × 20 × 120 mm) and repairs were performed with one (*n* = 6), or two (*n* = 6) 20 mm nitinol staples, or an eight-hole 2.7 mm quarter-tubular plate (*n* = 6). A pressure film was placed between fragments to determine contact area and compressive forces before and after loading. Loading consisted of multi-planar four-point bending with an actuator displacement of 3 mm. Gapping between segments was recorded to determine loads corresponding to a 2 mm gap and residual post-load gap.

**Results:**

Staple fixations showed statistically significant higher mean compressive loads and contact areas across the osteotomy compared to plate fixations. Double-staple constructs were superior to single-staple constructs for both parameters (*p* < 0.001). Double-staple constructs were significantly stiffer and endured significantly larger loads before 2 mm gap formation compared to other constructs in the dorsoventral plane (*p* < 0.001). However, both staple constructs were significantly less stiff and tolerated considerably lower loads before 2 mm gap formation when compared to plate constructs in the ventrodorsal and right-to-left lateral loading planes. Loading of staple constructs showed significantly reduced permanent gap formation in all planes except ventrodorsally when compared to plate constructs.

**Conclusions:**

Although staple fixations were not as stable as plate fixations in particular loading planes, double-staple constructs demonstrated the most consistent bending stiffness in all planes. Placing two perpendicular staples is suggested instead of single-staples whenever possible, with at least one staple applied on the compression side of the anticipated loading to improve construct stability.

## Background

The resultant rigidity and stability of internal fixation methods such as plates, screws, pins and cerclage wires have led to improved surgical and clinical outcomes. In recent years however, shape-memory alloy (SMA) staples have come to the forefront in the orthopaedics arena as alternative fixation devices.

SMA staples are often utilized clinically as fixation-compression devices for osteotomies, arthrodeses and fracture repairs especially of short bones, facial bones and the distal extremities (Choudhary et al. 2004; Laster et al. 2001; Neufeld et al. 2002; Rethnam et al. 2009). Yang et al. (1987) reviewed 51 cases where SMA staples were first used as an internal fixation device for a range of orthopaedic procedures and described good bone union and functional recovery in the 45 cases that were followed up. Similarly, a clinical study of tarsal joint arthrodesis with memory-compression staples undertaken by Malal, Hedge & Ferdinand (2006) also reported encouraging results of reduced time to fusion and improved post-operative recovery time. Reducing surgical trauma especially of the periosteum, is an important surgical consideration when choosing a preferred stabilisation technique for bone fixation due to its vital role in maintenance of the blood supply (Chao et al. 2012; Déjardin et al. 2012). From minimally invasive locking plates to intramedullary nailing, there are many options available to the surgeon (Perren 2002). Selection of implants is often subjective and dependant on the surgeon’s experiences or preferences. However, SMA staple application appears to have the benefit of being quicker and easier compared to other fixation types. The approach has been described as being simple and minimally invasive, which only involves making a small incision on the periosteum for holes to be drilled into each bone fragment prior to insertion of the staple (Singh et al. 2011). In Lapidus arthrodesis, for example, the incidences of non-union when employing nitinol staples have been shown to be similar to those reported for angle-stable internal fixation (Cottom and Vora 2013; Mallette et al. 2014), Clinically, this along with a shorter surgical and anaesthetic time potentially makes SMA staples a more favourable implant choice.

The most prevalent SMA for medical application is nitinol, a biocompatible nickel-titanium alloy with good corrosion resistance (Neufeld et al. 2002). Nitinol has two crystallographic phases, the strong higher-temperature austenite phase with a cubic crystalline structure and the malleable lower-temperature martensite phase with a twinned monoclonic structure (Ducheyne et al. 2011; Pfeifer et al. 2013). These states are often referred to as activated and non-activated and the temperature at which these phase changes occur can be influenced by design and manufacture (Mantovani 2000; Reedlunn et al. 2014). Previously investigated staples such as OSStaple™ (BioMedical Enterprises Inc, San Antonio, TX) and Memory Staple™ (DePuy Co., United Kingdom) (Rethnam et al. 2009; Shibuya et al. 2007), utilise the patient’s body temperature or external heating devices delivering bipolar electrical currents to initiate the shape-memory transformation (Ducheyne et al. 2011).

The staples investigated in the current study differ in that the transition temperature is set below room temperature and are therefore, active at room-temperature. These staples are supplied pre-loaded in an applicator which holds the staple legs open. The key characteristic of this nitinol staple exploits its superelasticity to constantly unload stress over great strains (Duerig et al. 1999), bringing with it the potential to provide immediate and sustained active compression which may help accelerate bone healing by closing any gap present or formed including those produced by osteoclastic resorption (Yang et al. 1987). This is in contrast to static compressive fixation methods, such as lag screws, which may lose the initial compression generated over time due to the viscoelastic relaxation of bone or if osteoclastic resorption occurs (Aiyer et al. 2016; Perren 2002; Yakacki et al. 2011).

There is a huge potential for the employment of nitinol staples in the orthopaedic field, but there is limited research available on staple use and how it compares to the current implants available as a form of fixation. Various studies report the use of two staples applied orthogonally to each other as a technique to increase the stability of the staple fixation (Bechtold et al. 1993; Choudhary et al. 2004). While these clinical studies have shown promising results, there has been little research investigating the actual biomechanics of the interface as dictated by this staple orientation. Likewise, the technique of orthogonal staple application has yet to be appraised against single-staple application, especially on the basis of interfragmentary compression and stability of the fixation. Buttress plating, a common technique for rigid fixation, was chosen as a control for the experiment as a basis of comparison to the staples. Hence, the purpose of this study is to biomechanically compare and contrast the three fixation methods of a single nitinol staple, orthogonally-applied double-staple and plate techniques across a simulated osteotomy site.

## Methods

### Study setting

The implants used were SMA staples (Speed™, BME Inc., San Antonio, Texas) with a bridge width and leg length of 20 mm and a cross-sectional profile of 2 mm by 2 mm. Upon release from the applicator, there is bridge-closure of 1.5 mm with a maximum closure of up to 10.8 mm at the leg extremities. An eight-hole 2.7 mm quarter-tubular bone plate with 2.7 × 22 mm self-tapping cortical bone screws (Synthes GmbH, Oberdorf, Switzerland) was used for comparison.

**Fig. 1:**
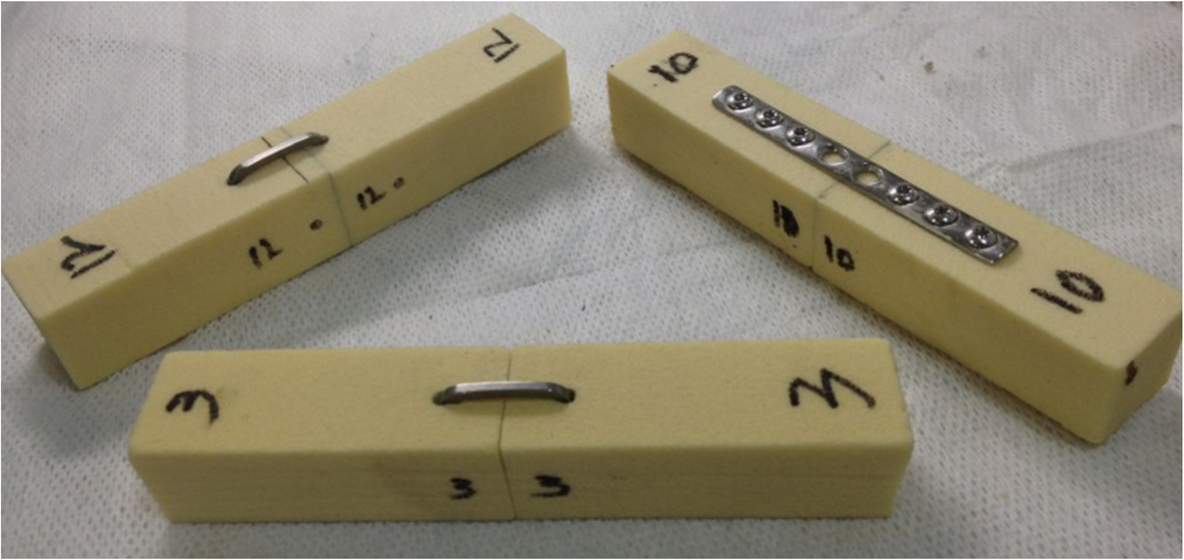
The dorsal surface is marked by the numbering of both far ends. The left-lateral surface is identified by the labelling adjacent to the planned osteotomy on the perpendicular surface. Ventral and right-lateral surfaces are opposite to the respective labelled surfaces previously described

A rigid closed-cell polyurethane (PU) foam of density 0.24 g/cm^3^ (Last-A-Foam® FR3700, General Plastics Manufacturing Co., Tacoma, WA), was selected as the surrogate material for the experiment, where 18 blocks with dimensions 20 × 20 × 120 mm were machined. These blocks were numbered and labelled as described in Fig. 1 to identify individual surfaces, determine planar orientation and avoid potential mismatching post-osteotomy. For the purposes of this study, the dorsal plane was deemed to be the plane on which the primary implant rests. A complete transverse osteotomy was created in the middle of each foam block. Six sets of the osteotomized polyurethane blocks were then randomly placed into each of the three groups for the experiment.

For single-staple constructs, 2.5 mm holes were pre-drilled using a guide and the nitinol staples loaded in the applicator were inserted into these holes and released. As for double-staple constructs, care was taken to avoid drilling into the perpendicular holes. Instead of drilling 10 mm on either side of the osteotomy as in the single staple construct, the drill holes were offset by 5 mm in opposing directions for each staple (Fig. 1). Plates were implanted by holding the centralised plate and synthetic block pieces flush with a bench-top vice while 2.0 mm pilot holes were drilled followed by the screws. Six screws were placed, leaving the two holes directly adjacent to the osteotomy open (Fig. 1). There was a sufficient quantity of plates and staples such that each plate and staple was used once.

Radiographs of all samples were taken before and after loading to objectively assess for the presence of plastic deformation of the implants or post-loading gap formation. All procedures were carried out at room temperature by the same investigator.

### Interfragmentary compression testing

A calibrated pressure film (Sensor model 5051, Tekscan Inc.) was inserted from the ventral surface between the osteotomized fragments prior to fixation of the implants. Interfacial forces were recorded from 5 seconds after completion of the constructs to reduce documenting any initial noise and axial forces placed by the investigator. Contact area, pressure maps and compressive forces were tracked using the I-Scan software (Tekscan Inc., South Boston, MA) for 10 min. This process was repeated after mechanical loading of the constructs.

### Mechanical testing

Constructs were loaded in four-point bending with an upper span of 30 mm and a lower span of 90 mm in a Material Testing System (MTS) Mini Bionix 858 test frame (MTS Systems, Eden Prairie, MN). The specimens were subjected to a displacement-controlled test at 1 mm/min until axial deflection of the actuator reached 3 mm before returning back to zero. Loading was conducted on the equivalent of dorsoventral, right-to-left lateral and ventrodorsal planes. Left-to-right lateral loading was also performed for all constructs as the double-staple construct was not symmetrical. The order in which the planar testing was performed was kept consistent for all of the testing. This took into account the plastic deformation that occurred in the plate tent groups so that the dorsoventral test was performed last. Time, load and displacement data was recorded at 100 Hz and used to calculate stiffness and peak loads.

Imaging of the osteotomy site was synchronised with the four-point bending test procedure using a Canon EOS 700D at 0.2Hz. Images were calibrated and analyzed with ImageJ 1.47 t (National Institutes of Health, USA) to measure gapping between the distal ends of the osteotomy. This was matched to the load data to determine loads required to produce a 2 mm gap. Permanent gap formation after loading was similarly measured with the imaging software by comparing any gap difference in the constructs pre and post-loading.

### Statistical analysis

A multivariate general linear model with post hoc comparisons was used to compare the outcomes of mechanical testing, mean compressive force and mean area of compression of the three groups (IBM SPSS Statistics Version 21, IBM Corp.). Two-tailed paired *t*-test was also performed to evaluate the difference between compressive force and area across the osteotomy initially and after 10 min. Mean compressive force and area of compression before and after loading were evaluated similarly. P value <0.05 was considered to be significant.

## Results

### Interfragmentary compression

**Fig. 2:**
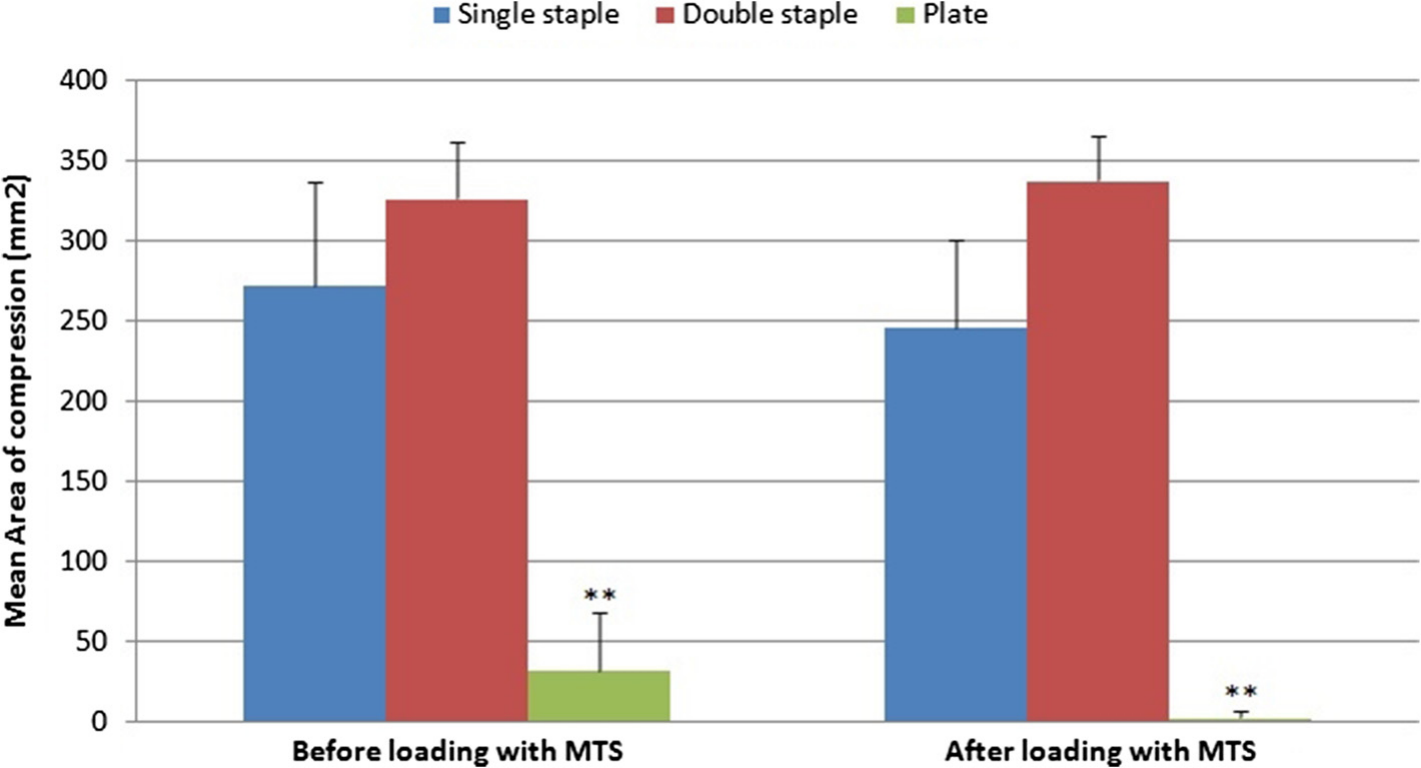
Mean area of compression before and after loading. **compared to the other constructs (*p* < 0.001)

The mean area of the compression footprint across the osteotomy by a plate construct was found to be 8.42 and 10.1 times less than single-staple and double-staple fixations respectively (*p* < 0.001) (Fig. 2 the total area of contact ideally being 400 mm^2^−20 mm × 20 mm). Although double-staple constructs (326.0 ± 63.86 mm^2^) showed a higher mean area of compression compared to single-staple fixations (272.17 ± 63.86 mm^2^), this was not statistically significant. The area of compression before and after loading also showed no significant difference for single-staple, double-staple and plate constructs.

Mean compressive force for single nitinol staple constructs, 36.6 ± 8.0 N, was greater than that of plate fixations, 2.73 ± 3.09 N (*p* < 0.001). Double-staple fixation, however, showed significantly greater mean compressive forces than single staples (*p* < 0.001), with double-staple constructs 29.0 times greater than plates. Single-staple (32.0 ± 4.99 N), double-staple (74.3 ± 4.83 N) and plate constructs (0 ± 0) all displayed a non-significant decrease in compressive forces post-loading.

**Fig. 3:**
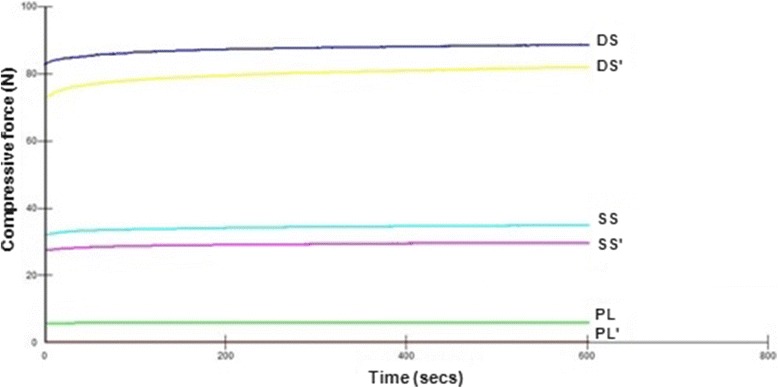
Compressive force over 10 min constructs before and after loading. SS: Single-staple, DS: Double-staple, PL: Plate. The post-loading data are indicated by SS’, DS’, PL’ respectively

Interfragmentary compressive forces for staple constructs were found to have increased over time as displayed in Fig. 3. Initial compressive force across the osteotomy for single and double-staple constructs increased from 34.60 ± 7.55 N to 75.53 ± 8.66 N respectively, to 37.18 ± 8.07 N and 80.48 ± 9.63 N after 10 min (*p* < 0.001). However, the compressive forces for plate constructs initially (2.70 ± 3.01 N) and after 10 min (2.75 ± 3.11 N) were not found to be statistically different. Interfacial area of compression also showed a similar increase from 272.0 ± 69.83 mm^2^ to 314.5 ± 43.97 mm^2^ at the start for single and double-staple constructs respectively, to 274.67 ± 66.63 mm^2^ and 324.33 ± 35.87 mm^2^ after 10 min. However, neither these results nor the difference in area of compression across the osteotomy for plate constructs were statistically different.

**Fig. 4:**
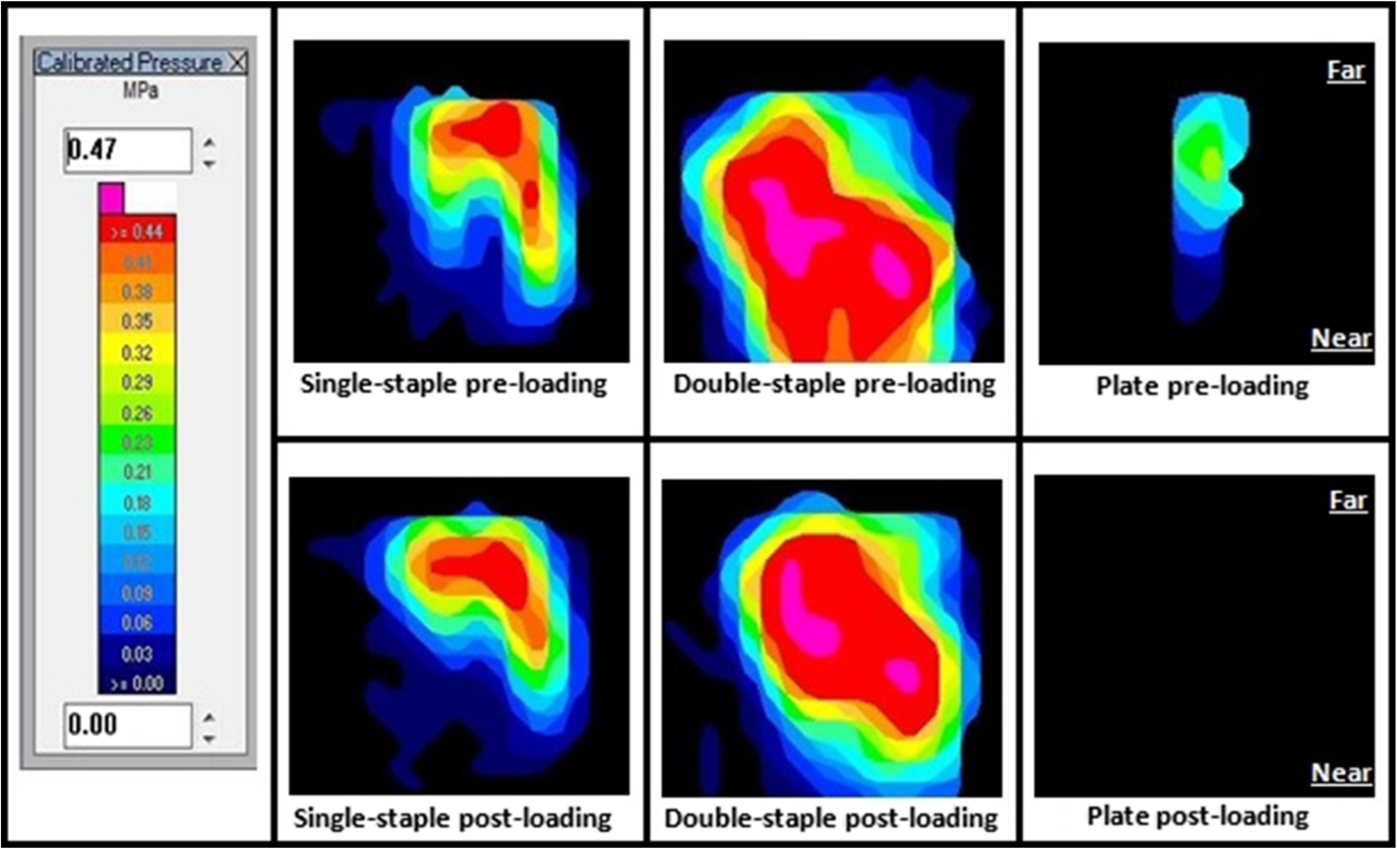
Example of pressure distribution across the osteotomy. Legend (left) shows scale of pressure intensity. Far and near sides of the osteotomy are also indicated

Qualitative assessment of the pressure maps (Fig. 4) showed moderate to marked contact pressure applied on the far end of the osteotomy in single-staple constructs, with moderately weaker contact pressure on the near end in comparison. A similar appearance was noted post-loading. With double-staple constructs, however, contact pressure was noted to be more evenly distributed between near and far ends of the osteotomy. A drop was noticed at the near end of the osteotomy after loading with the MTS but the resultant contact pressure was still considerably greater than single-staple constructs. Comparatively, a small area of mild pressure was shown for plate fixations and all contact pressure was lost upon loading with the MTS.

### Mechanical testing

Loading of the double-staple constructs resulted in material shearing failure in two out of six samples during mechanical testing. One occurred during loading in left-to-right lateral plane and the other in the ventrodorsal plane. Data obtained from both tests were deemed invalid and omitted in the tabulation of results.

**Fig. 5:**
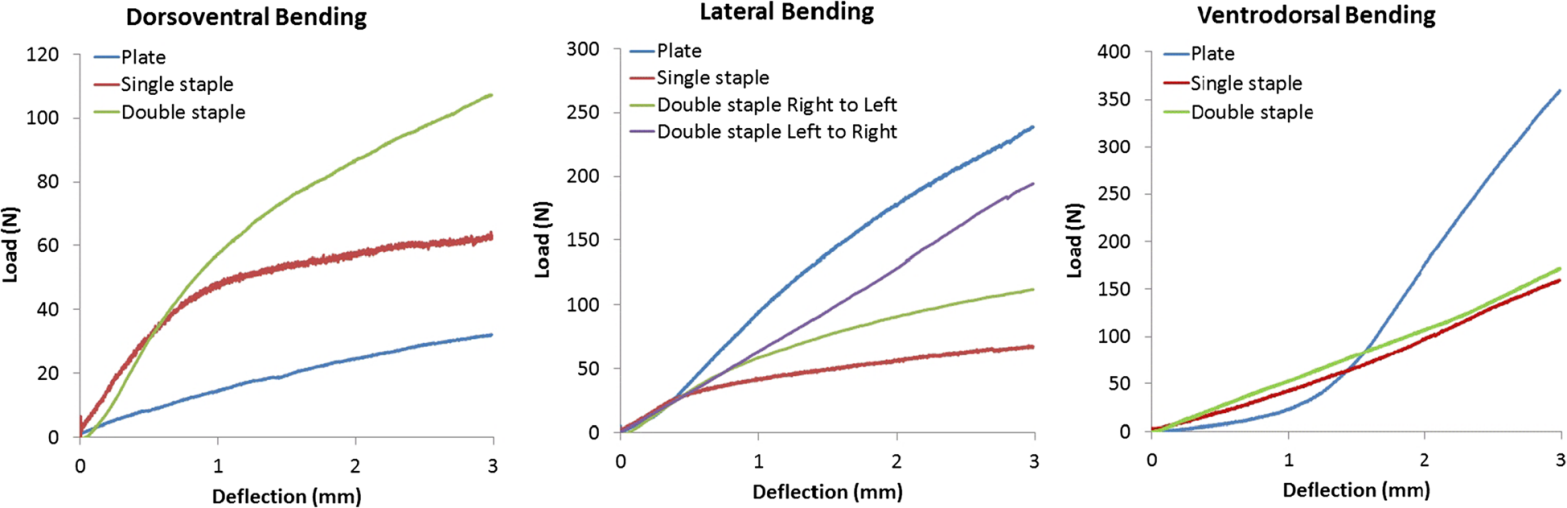
Load-displacement curves in dorsoventral, lateral and ventrodorsal planes

Double-staple constructs (29.0 ± 5.12 N/mm) were found to be the most rigid fixation in the dorsoventral plane, being 3.13 and 2.68 times stiffer compared to single-staple and plate constructs respectively (*p* < 0.001) (Fig. 5). No statistical differences were detected between plate (10.81 ± 2.73 N/mm) and single-staple constructs (9.27 ± 1.27 N/mm) in this plane.

Plate constructs (73.99 ± 4.61 N/mm) in right-to-left lateral four-point bending were found to be significantly more rigid in this plane compared to single and double-staple constructs, being 5.09 and 2.49 times stiffer respectively (*p* < 0.001). However, despite the difference suggesting plates being stiffer than double-staple constructs in the left-to-right lateral plane, this difference was not statistically significant. Double-staple fixations were also noted to be significantly stiffer in the right-to-left lateral (29.70 ± 4.84 N/mm) and left-to-right lateral planes (63.73 ± 8.79 N/mm) compared to single-staple fixations (14.53 ± 2.38 N/mm) (*p* < 0.001). Four-point bending of the constructs in the ventrodorsal plane showed that plate fixation (152.64 ± 50.48 N/mm) was stiffer compared to double (57.40 ± 6.76 N/mm) and single staples (61.76 ± 10.89 N/mm) (*p* < 0.001). The stiffness of double-staple constructs, however, was not statistically different to single-staple constructs in this plane.

**Fig. 6:**
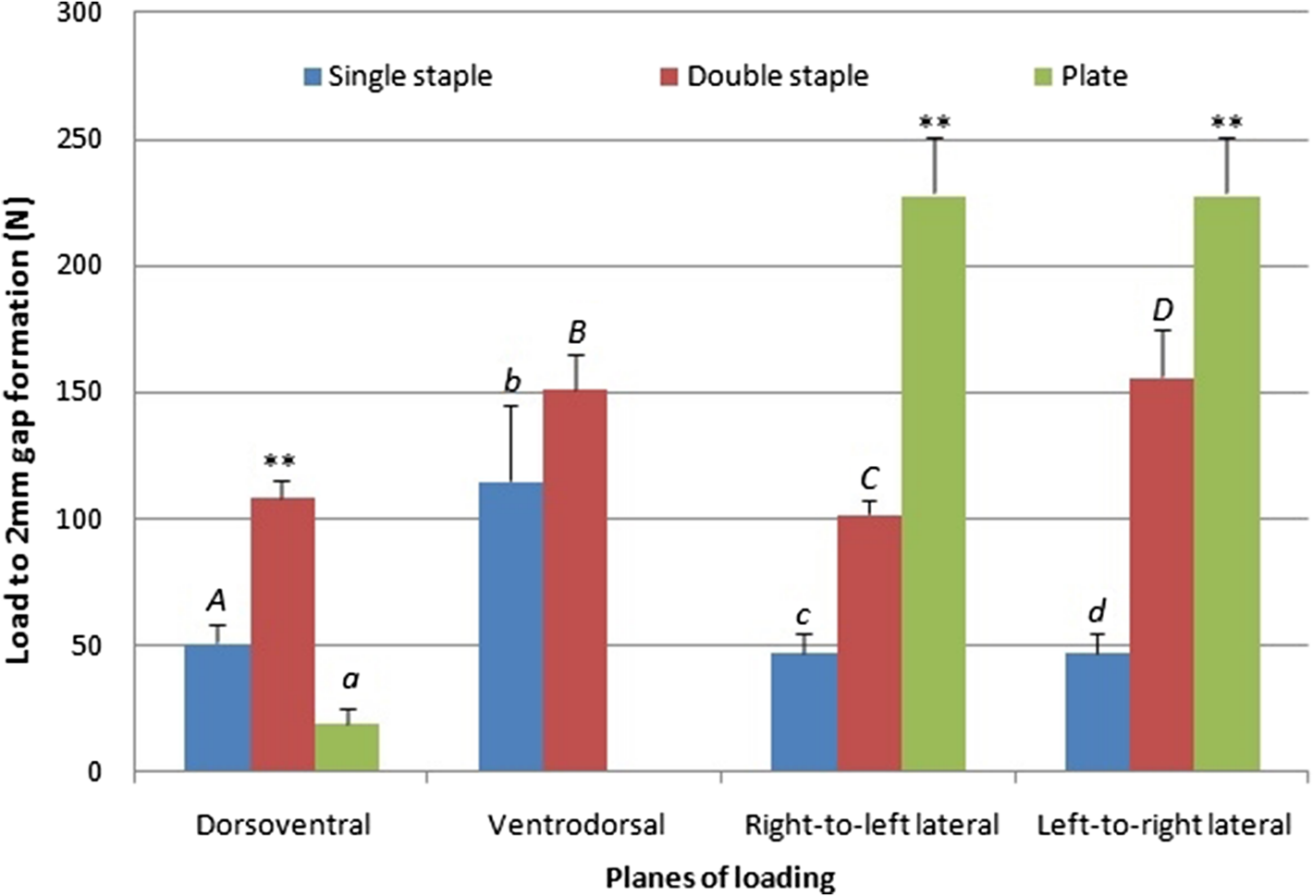
Loads at which a 2 mm gap formed in each construct/loading plane. **compared to others in the same plane (*p* < 0.05). Capital and lower cases signify statistical difference between two constructs in the same plane (*p* < 0.05)

In the dorsoventral loading plane, double-staple constructs were found to bear significantly larger loads of 108.42 ± 7.13 N before 2 mm gap formation compared to plate (19.50 ± 6.16 N) and single-staple constructs (51.10 ± 7.15 N) (*p* < 0.001) (Fig. 6). Interestingly, single-staple constructs were seen to be able to withstand 2.62 times more load compared to plate fixations before 2 mm gapping occurred during four-point bend in this plane (*p* < 0.001). Plate constructs (228.49 ± 22.65 N) were able to withstand a significantly higher load compared to single (47.28 ± 7.55 N) and double-staple constructs in the left-to-right (156.14 ± 18.59 N) and right-to-left lateral planes (102.0 ± 5.50 N) before exhibiting 2 mm gap formation at the osteotomy site (*p* < 0.001). Double-staple constructs were also found to bear a significantly larger load before 2 mm gapping in both left-to-right and right-to-left lateral planar four-point bending compared to single-staple constructs (*p* < 0.001). All plate constructs loaded ventrodorsally failed to reach a 2 mm gap despite the 3 mm displacement-controlled axial loading, indicating enhanced construct stability compared to the staple fixations. To form a 2 mm gap across the osteotomy in double-staple constructs during ventrodorsal four-point bending, a load 1.38 times greater than that required for single-staple construct (114.82 ± 29.71 N) was required (*p* = 0.021).

**Fig. 7:**
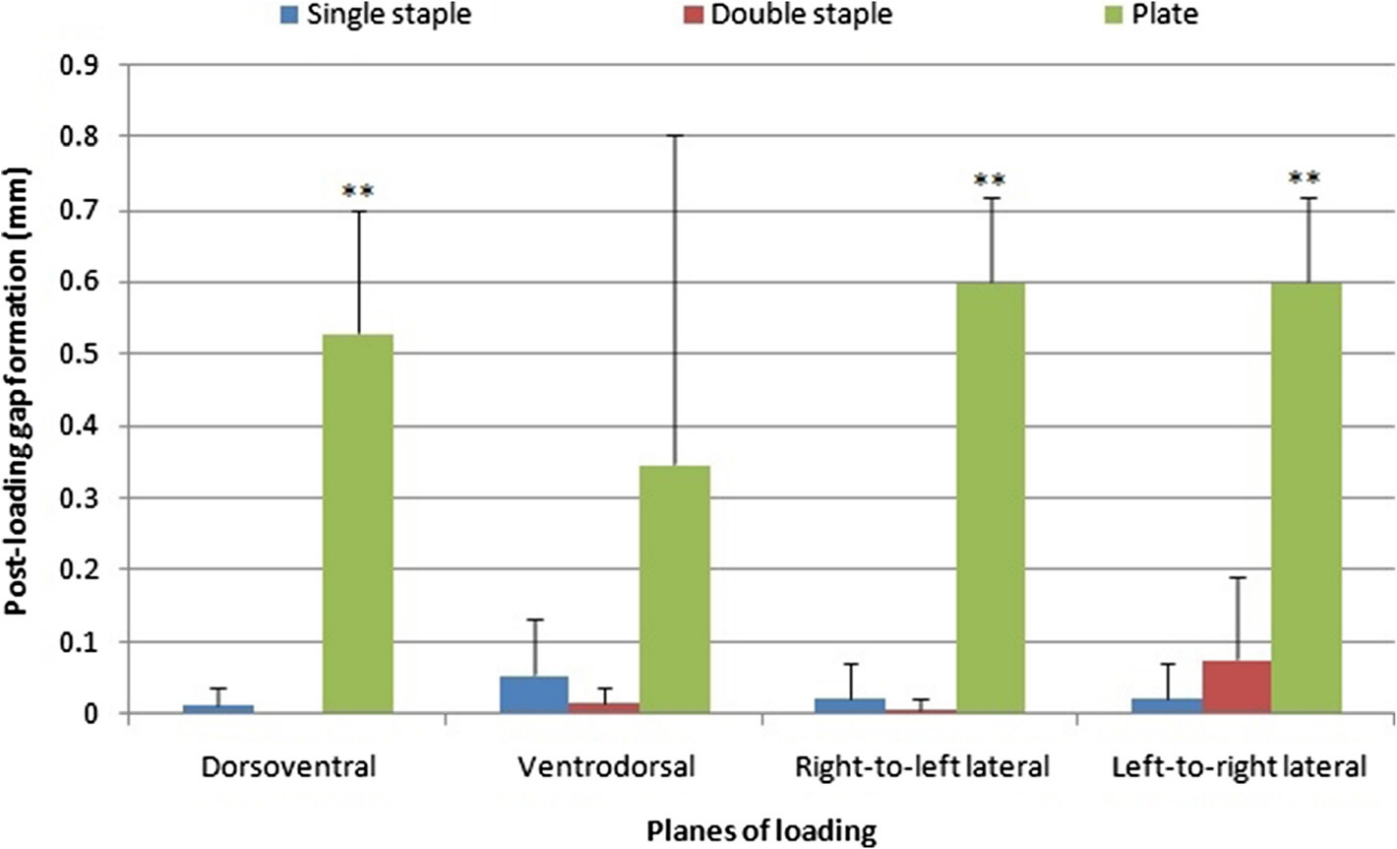
Residual gap post-loading of fixations in all 4 planes. **compared to the other two constructs in the same plane (*p* < 0.05)

**Fig. 8:**
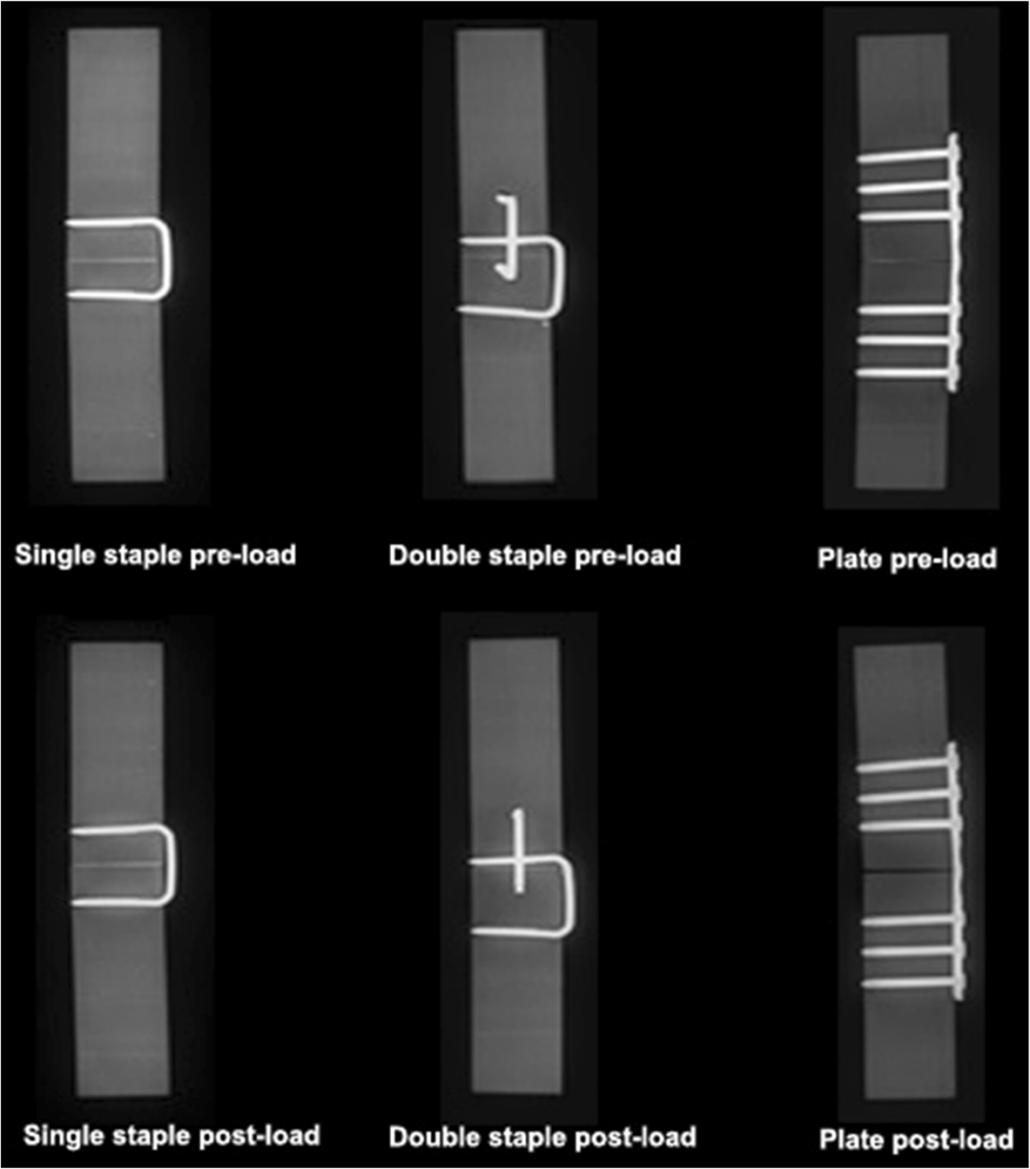
Examples of lateral radiographs taken of the constructs pre and post-loading. Subjective assessment shows consistent gap formation of plate constructs after loading compared to minimal to no gap formation in staple constructs after loading

Plate fixations (0.53 ± 0.17 mm) developed larger permanent gap formation compared to both staple fixations after dorsoventral loading (*p* < 0.001) (Fig. 7). No difference was detected in gap formation post-loading for single (0.011 ± 0.027 mm) and double-staple constructs (0 ± 0 mm) in this plane. Following loading in right-to-left lateral plane, plate constructs (0.60 ± 0.12 mm) displayed larger permanent post-loading gap compared to double and single staples by 0.63 and 0.62 mm respectively (*p* < 0.001). Permanent gap in plate fixations was also considerably larger compared to double-staple constructs loaded in left-to-right lateral planes (*p* = 0.03). No difference in post-loading gap was found between single (0.020 ± 0.049 mm) and double-staple constructs in the left-to-right (0.075 ± 0.11 mm) (*p* = 0.900) or right-to-left lateral plane (0.007 ± 0.016 mm) (*p* = 0.907). The findings of post-loading gap in the three constructs coincide with the radiographic appearance (Fig. 8). Permanent gap formation was not different for the plate constructs (0.35 ± 0.46 mm) single (0.054 ± 0.079 mm) or double staples (0.014 ± 0.022 mm) in the ventrodorsal plane (*p* > 0.73).

## Discussion

The present study evaluated plate fixation versus SMA staple fixation of osteotomies in a highly reproducible model that investigated gap formation, interfacial pressure and area, as well as construct stiffness. PU foam, designed to simulate cancellous bone, was used to decrease the variability and allow the performance of the repair to be evaluated without external variation. Short bones such as carpal and tarsal bones, the main targets for the SMA staple fixation in human fracture scenarios, consist primarily of cancellous bone and as such make PU foam an ideal candidate for clinical replication (Bechtold et al. 1993; Qin 2013; Rovinsky et al. 2000).

The nitinol staple constructs in this study have demonstrated markedly higher compressive area and load through their shape-memory and superelasticity properties as compared to the mild compression that plate constructs provide. These results are consistent with other studies evaluating the benefits of SMA implants (Aiyer et al. 2016; Kildow et al. 2016; Russell et al. 2015). In a biomechanical study of a tarsometatarsal model comparing SMA staple fixations against plate or screw fixations, Aiyer et al. (2016) similarly reported a significantly greater contact profile and maintenance of contact area post-loading of SMA staple fixations compared to the rest. It was remarkable to note in our study that doubling the staple implants and applying them orthogonally resulted in an increase in compressive forces by approximately 2.16 times compared to single-staple application. Generating compression across a fracture surface stabilises the fracture site by increasing the frictional contact between the opposed bone ends to neutralize micromotion and withstand bending, shearing & torsional stressors (Ashhurst 1986; Kaspar et al. 2005). Rigid compression fixations with minimal interfragmentary movement as such, has been shown to be beneficial for primary bone healing (Chalayon et al. 2015; Latt et al. 2015; Mirhadi et al. 2013). Promoting direct bone healing is especially critical to achieve successful union of ankle arthrodeses due to the lack of periosteal or endosteal joint coverage to facilitate secondary healing via callus formation (Kildow et al. 2016; Yakacki et al. 2011).

In this study, single and double nitinol staples have also demonstrated a “dynamic active compression” property with both displaying an approximate 7 % increase in interfragmentary compressive loads across the simulated osteotomy over 10 min. In comparison, no such change in compressive force was identified from the static fixation of plate constructs over time. The same pattern of increasing compression over time was also seen in the post-loading staple fixations (Fig. 3). This ability to maintain and adapt to slight changes in position to maintain compression across an osteotomy was demonstrated by the staples via similar pre-loading and post-loading pressure profiles as displayed in Fig. 4 and the minimal permanent post-loading gap formation measured. This was especially evident with the double-staple constructs and is likely to be attributed to the higher interfacial forces and synergistic compression applied in perpendicular planes. In contrast, a minor non-return gapping of 0.05 mm was noted after ventrodorsal loading of single-staple constructs. Although this finding was not found to have a statistically significant difference when compared to the other constructs in the same loading plane, this small gap formation could be attributed to the reduced interfragmentary compression proximal to the nitinol staple legs. Comparatively, a significantly higher permanent gap formation of approximately 0.5 mm in all other loading planes was noted in plate constructs. This was postulated to be due to their lack of continual compression as well as the plastic deformation resulting in the loss of the initial static compression. The effect of such gap formations across the osteotomy clinically would potentially be the onset of slower secondary healing rather than the faster primary contact healing process (Augat et al. 1998; Chao et al. 2012; Lutz et al. 1997).

These findings of dynamic compression mirror those of other studies and support the use of shape memory staples in clinical settings to counteract bone resorption that occurs during the natural healing process and sustain compression (Aiyer et al. 2016; Kildow et al. 2016; Yakacki et al. 2011). Non-union of ankle arthrodesis in humans have been described to be between 9 and 17 %, with bone resorption attributed to be a major contributing factor especially in static fixation constructs (Chalayon et al. 2015; Latt et al. 2015). This is supported by a study conducted by Yakacki et al. (2010) that demonstrated the inability of static internal fixations to provide sustained compression in the face of bone resorption. Intramedullary nail fixations in both synthetic and cadaveric constructs demonstrated a 90 % reduction in compression upon resorption of less than 1 mm (Kinmon et al. 2013; Yakacki et al. 2010). The loss of stabilising compression as a result of the natural bone end resorption leads to increased micromotion and gap formation. Hence, the resultant local instability will stimulate further bone resorption which can potentially compound a negative cycle causing increasing construct instability and ultimately, non-union or hardware failure (Kinmon et al. 2013; Yakacki et al. 2010).

Stability and strength of the constructs in the different planes of loading were determined based on their respective stiffness and loads tolerated before 2 mm gap formation. Double-staple constructs demonstrated the most consistent bending stiffness in all planes. Besides the comparable stability under the ventrodorsal loading, double-staple constructs also displayed significantly superior bending rigidity to single-staple constructs in all other loading planes. However, the results of this study indicate that plate fixations are significantly stiffer constructs as compared to staple fixations when loaded in the ventrodorsal and right-to-left lateral planes. The stability of these constructs in these planes was similarly mirrored in the findings for load required to elicit a 2 mm gap. The increased rigidity of plate constructs in these planes can be attributed to their greater area moment of inertia. Area moment of inertia, the geometrical capability of an object to resist bending upon mechanical loading, is primarily determined by an object’s cross-sectional profile and the direction of applied load (Chao et al. 2012; Wilcox 2006). Hence, the larger rectangular geometry of plates compared to the smaller cross-sectional profile of staples result in a greater mass distribution away from the neutral axis and consequently, increases the area moment of inertia and general stability of the construct (Chao et al. 2012; Wilcox 2006).

The bending rigidity of the single-staple constructs under dorsoventral loading, however, were shown to be comparable to plate constructs with the former also showing the capacity to withstand 2.62 times more load compared to the latter before 2 mm gapping occurred. Double-staple constructs were also found to be the most rigid fixation in this loading plane, being 2.68 times stiffer than plate constructs. Four-point bending via the MTS machine acts to place the top surface of the constructs under compressive stressors and the bottom surface under tensile stressors. Hence, the interfragmentary forces elicited by the nitinol staples could play a role in enhancing bending resistance through load-sharing where part of the load is redirected across the compressed osteotomy line (Bucholz 2010). With staple compression especially focused across the far end of the osteotomy as depicted on the pressure maps (Fig. 4), the tensile forces of bending are also concurrently neutralized. In comparison, load application with a lack of compression in the plate constructs results in all forces transmitted entirely through the implant alone where the full bending forces are subjected at the point of the fracture gap as its fulcrum (Bucholz 2010). These results may potentially assist surgeons in selecting the best area to place the respective implants to maximise the bending rigidity in the specific plane of interest. Furthermore, the enhanced compression and stability elicited by double-staple constructs in this study would advocate for placing two nitinol staples in an orthogonal fashion rather than a single staple as a preferable option for fixation whenever possible.

### Limitations

Using compression plating would have been the ideal comparison for the staple constructs based on function and may provide a different contact profile to the shape-memory alloy staples before and after loading and we would recommend this for future work. Despite the promise of double nitinol staples applied orthogonally as a form of internal fixation, the study is limited as the influences of an in vivo environment such as soft tissues and ligamentous constraint (Chao et al. 2012; Shibuya et al. 2007) were not fully considered. The stress relaxation properties of bone act to limit the maximum compression force of the implant post-application (Quaglini et al. 2009; Yakacki et al. 2010). Bone is subjected to axial, bending, shear and torsional loads and the fixation chosen for repair must be able to withstand these forces in order to avoid construct failure (Epari et al. 2010). All other loading forces besides bending were not examined in the current study. Until these are evaluated, it is difficult to comment if the staple fixations would allow for earlier resumption of post-operative weight bearing (Prissel et al. 2016). The two constructs that failed were excluded from the study and thus decreased the statistical power of the study. These failures appeared to be the result of placing the second of the two staples in the double staple group too close to the osteotomy site where the staple arm pulled through the foam; this stands as an important point to remember when using a double staple technique clinically. Other areas of interest that are recommended for future research include additional staple orientations (Bechtold et al. 1993) and the impact of micro-motion on these fixation constructs.

## Conclusion

SMA staples used in this study are distinguishable from plate implants via their markedly higher mean compressive forces and area of compression across the osteotomy. Moreover, these staples were found to exhibit dynamic active compression where compression was not lost after loading but was seen to have increased over time instead. Double-staple constructs exhibited the most consistent bending stability in all planes, while the plate fixation produced both the most and least stiff construct depending on bending plane. The results of this study should be beneficial in appreciating the biomechanics of nitinol staples and their relevant impacts depending on the method of application. The findings of this study emphasise the importance of understanding the expected post-operative loading of bone to better place orthopaedic implants to assist bone healing. It is therefore suggested that two nitinol staples be placed in an orthogonal fashion in preference to a single-staple whenever possible with at least one staple to be placed on the compression side of the anticipated loading to maximize construct stability. While many methods of fracture fixation are currently available to surgeons, choosing an appropriate technique comes down to what the individual surgeon is trying to achieve biomechanically and physiologically. The use of plates for small bone fractures requires relatively more hardware to local tissue than staples. Greater time and effort is required to apply these plates. The use of lag screws requires accurate placement to achieve a good quality compressive force across the fracture site. Hence, the use of a single or double staple technique for the appropriately chosen clinical situation such as carpal, tarsal, metacarpal and metacarpal bones appears to be a viable alternative fixation method, either alone or in combination with plates and or screws. The significance of these findings should be further evaluated through in vivo studies to assess its validity in clinical applications.

## Abbreviations

DD: double staple
MANOVA: multivariate analysis of variance
MTS: material testing system
PU: polyurethane
SMA: shape memory alloy
SS: single staple
